# Caspase-3 mediated release of SAC domain containing fragment from Par-4 is necessary for the sphingosine-induced apoptosis in Jurkat cells

**DOI:** 10.1186/1750-2187-8-2

**Published:** 2013-02-27

**Authors:** Faisal Thayyullathil, Siraj Pallichankandy, Anees Rahman, Jaleel Kizhakkayil, Shahanas Chathoth, Mahendra Patel, Sehamuddin Galadari

**Affiliations:** 1 Cell Signaling Laboratory, Department of Biochemistry, College of Medicine and Health Sciences, UAE University, P.O. Box 17666, Al Ain, Abu Dhabi, UAE

**Keywords:** Par-4, Caspase, Apoptosis, SAC domain, Cleavage, Sphingosine

## Abstract

**Background:**

Prostate apoptosis response-4 (Par-4) is a tumor-suppressor protein that selectively activates and induces apoptosis in cancer cells, but not in normal cells. The cancer specific pro-apoptotic function of Par-4 is encoded in its centrally located SAC (Selective for Apoptosis induction in Cancer cells) domain (amino acids 137–195). The SAC domain itself is capable of nuclear entry, caspase activation, inhibition of NF-κB activity, and induction of apoptosis in cancer cells. However, the precise mechanism(s) of how the SAC domain is released from Par-4, in response to apoptotic stimulation, is not well explored.

**Results:**

In this study, we demonstrate for the first time that sphingosine (SPH), a member of the sphingolipid family, induces caspase-dependant cleavage of Par-4, leading to the release of SAC domain containing fragment from it. Par-4 is cleaved at the EEPD131G site on incubation with caspase-3 *in vitro,* and by treating cells with several anti-cancer agents. The caspase-3 mediated cleavage of Par-4 is blocked by addition of the pan-caspase inhibitor z-VAD-fmk, caspase-3 specific inhibitor Ac-DEVD-CHO, and by introduction of alanine substitution for D131 residue. Moreover, suppression of SPH-induced Akt dephosphorylation also abrogated the caspase dependant cleavage of Par-4.

**Conclusion:**

Evidence provided here shows that Par-4 is cleaved by caspase-3 during SPH-induced apoptosis. Cleavage of Par-4 leads to the generation of SAC domain containing fragment which may possibly be essential and sufficient to induce or augment apoptosis in cancer cells.

## Background

Programmed cell death or apoptosis, plays an important role in biology; such as differentiation, control of cell number, and removal of damaged cells. It is also implicated in cancer, autoimmune and neurodegenerative diseases [1,2]. Apoptosis is characterized by the activation of multifunctional, highly regulated family of cysteine-dependent aspartate-directed protease enzymes called caspases. These enzymes catalyze biologically diverse set of reactions and play a critical role in the initiation and execution of apoptosis. The activation of initiator caspases (caspase-8 and −9) results in the cleavage and activation of the downstream effector caspases (caspase-3, -6, and −7). The downstream caspases are in turn responsible for the selective and limited proteolysis of multiple cellular proteins involved in the morphological and biochemical changes that are associated with apoptosis [3]. A large number of substrates for caspases have already been identified. These include structural proteins such as nuclear lamins, proteins involved in the DNA repair mechanism such as poly (ADP-ribose) polymerase (PARP), and tumor suppressor protein such as p53 [4,5].

Prostate apoptosis response-4 (Par-4), the product of the proapoptotic gene *par-4* was first identified in prostate cancer cells when they were induced to undergo apoptosis [6]. Human Par-4 is a 340-amino acid protein having an apparent molecular weight of about 40 kDa, with a leucine zipper domain (amino acids 290–332) at the carboxy terminal end [7]. Par-4 has two putative nuclear localization sequences namely NLS1 (amino acids residues 20–25) and NLS2 (a bipartite sequence comprising amino acid residues 137–153). Removal of NLS1 by deletion of the first 68 amino acids did not affect the apoptotic function of Par-4 [7]. In contrast, deletion of NLS2 sequence abrogated the ability of mutant fragment to translocate to the nucleus. The NLS2 mutant Par-4 is also unable to induce apoptosis. The cancer selective apoptotic action of Par-4 is localized in its central core SAC (Selective for Apoptosis induction in Cancer cells) domain which comprises amino acids 137–195 [8]. Interestingly, SAC domain that contains NLS2 domain localizes to the nucleus in normal, immortalized, and cancer cells. The SAC domain is capable of inducing caspase activation, inhibition of Bcl-2 expression, and down-regulation of transcription factor NFκB. Moreover, the SAC domain itself induces apoptosis not only in Par-4 sensitive cancer cells, but also in cells that are resistant to full length Par-4 inducible apoptosis [8].

Sphingosine (SPH), a sphingolipid metabolite, has come to prominence as a bioactive lipid. Indeed, SPH has been shown to be a critical mediator in TNF-α-, Fas-, phorobol ester-, and doxorubicin-induced apoptosis in variety of cell types [9-11]. Additionally, exogenous SPH also induces apoptosis in different cancer cells [10,12,13]. However, despite the fact that SPH function as an important signaling molecule in the regulation of cell growth and apoptosis, its mechanism of action is still not clear. Interestingly, it has been reported that SPH down-regulates the expression of the anti-apoptotic protein Bcl-2 [14], activates various caspases and stimulates PARP cleavage, a well known target for caspases [15].

Akt (also known as protein kinase B; PKB), has been identified as a key downstream effector of phosphoinositide-3-kinase (PI3K) that blocks apoptosis in variety of cell types [16]. Akt is an inactive cytosolic protein, which is recruited to the plasma membrane and activated by phosphorylation at Thr^308^ and Ser^473^ in response to growth factors or cytokines [17,18]*.* Akt is known to prevent apoptosis by catalyzing the phosphorylation of a number of downstream targets including Par-4 [19,20], GSK-3β [21], BAD [22], caspase-9 [23], XIAP [24], ASK-1 [25], Mdm2 [26]. Following its activation, Akt is inactivated by dephosphorylation, which is mediated by protein phosphatae-1 (PP1) or protein phosphatase-2A (PP2A) like phosphatases [27,28]. Recently, we have shown that protein phosphatase-1-dependant inhibition of Akt phosphorylation is critical for the SPH-induced apoptosis in human leukemic cells [12].

Caspase-mediated cleavage of specific target proteins generally results in either activation of proteins that participate in the execution of apoptosis, or inhibition of target proteins that would normally promote cell survival [29]. The cleavage may cause a change in the function and/or localization of target proteins [3]. In this study, we demonstrate for the first time the selective caspase dependant release of SAC domain containing fragment from Par-4 during SPH-induced apoptosis. Additionally, we demonstrate the suppression of SPH-induced Akt dephosphorylation at Ser^473^with calyculin A and phosphatidic acid (PA) protected caspase-dependent cleavage of Par-4 and apoptotic signalling pathways in Jurkat cells.

## Results

### Par-4 is cleaved during SPH-induced apoptosis in Jurkat cells

**Figure 1:**
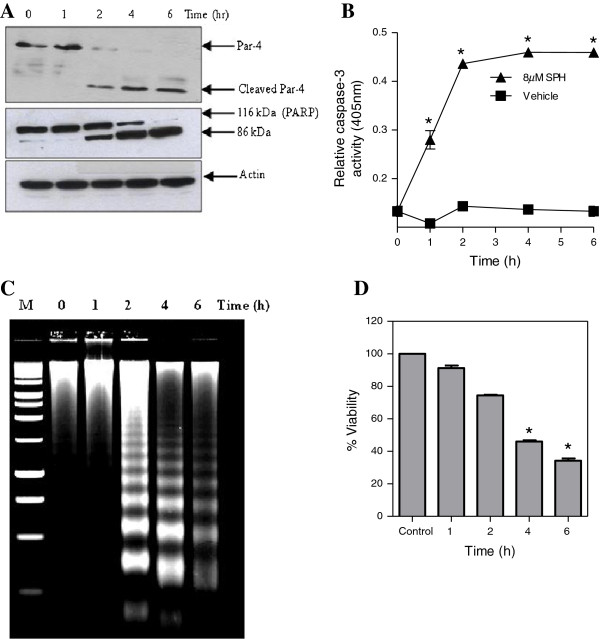
**Sphingosine induces Par-4 cleavage in Jurkat cells.** Panel **A***,* Jurkat cells were treated with 8 μM SPH for the indicated time period and western blot analysis of cell lysate was carried out using an antibody against Par-4. Apoptosis induction was confirmed by western blot analysis of PARP cleavage using an antibody recognizing full-length and cleaved form of PARP. Control of protein loading was confirmed by using an antibody against actin. Panel **B***,* Jurkat cells treated with SPH (8 μM) for the indicated time period and enzymatic activity of caspase-3 was measured as described in the materials and methods. Data shown are means ± SD (n = 3). *, *p* < 0.05 compared with untreated control. Panel **C***,* Jurkat cells were treated with SPH (8 μM) for indicated time period and DNA fragmentation was analysed as described in the materials and methods. Panel **D***,* WST cell viability assay for Jurkat cells following treatment with SPH (8 μM) for indicated time. Data shown are means ± SD (n = 3). *, *p* < 0.05 compared with untreated control.

Recently, Chaudhry and colleagues demonstrated that Par-4 is a novel substrate for caspase-3 during cisplatin-induced apoptosis in ovarian cancer cells [30]. In this study, we sought to extend this observation and to determine whether activation of the caspase cascade induced by SPH, plays a role in the cleavage of Par-4 protein in Jurkat cells. Exposure of Jurkat cells to 8 μM SPH for 6 h decreased the level of intact Par-4 protein, and generated a truncated form of Par-4 which migrates at approximately 24 kDa based on western blot analysis. The Par-4 antibody used in this experiment was raised against the C-terminal epitope, and hence, the recognized band is the C-terminal fragment of Par-4 protein. Cleavage of Par-4 was first detected 2 h after the addition of SPH and completed within 6 h (Figure 1A, *top panel*). Importantly, Par-4 cleavage is an early event during apoptosis, and it occurs with a similar time course to the PARP cleavage (Figure 1A, *middle panel*), caspase activation (Figure 1B), DNA fragmentation (Figure 1C), and loss of viability (Figure 1D); four distinct features of cells undergoing apoptosis.

### Par-4 cleavage is a universal step in caspase-dependant apoptosis

**Figure 2:**
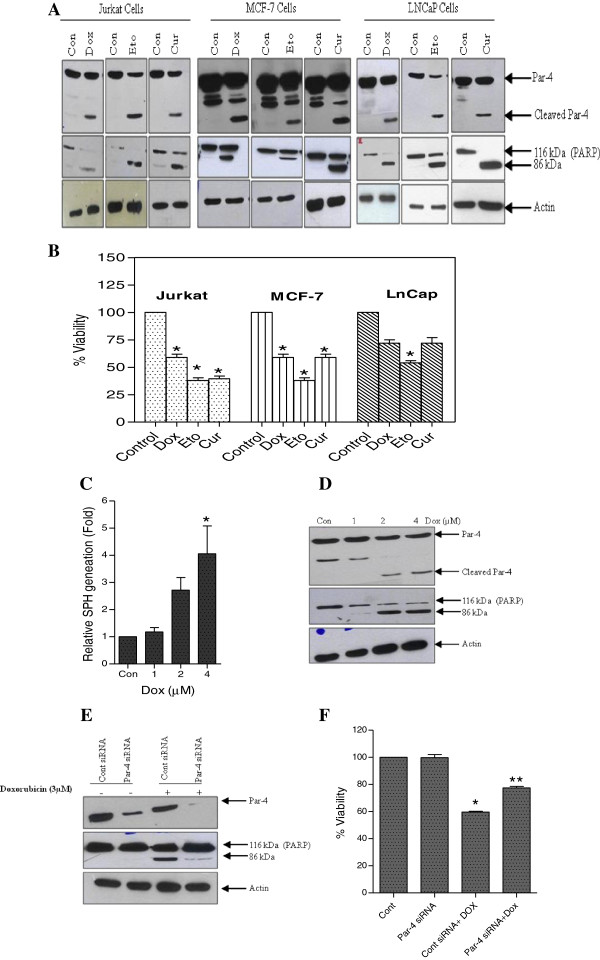
**Par-4 cleavage is a common step in the process of caspase-dependant apoptosis.** Panel **A***,* Jurkat cells (0.2 μM Dox, 4 μM Eto and 25 μM Cur), MCF-7 cells (3 μM Dox, 100 μM Eto and 40 μM Cur) and LNCaP cells (5 μM Dox, 50 μM Eto and 50 μM Cur) were treated for 24 h and cleavage of Par-4 was analyzed by western blot analysis using Par-4 antibody. Apoptosis induction was confirmed by PARP cleavage and actin was used as protein loading control. Panel **B***,* Jurkat, MCF-7 and LNCaP cells were treated with Dox, Eto and Cur for 24 h and cell viability was measured by using WST viability assay kit. Data shown are means ± SD (n = 3). *, *p* < 0.05 compared with untreated control. Panel **C***,* MCF-7 cells were treated with indicated concentration of Dox and intracellular SPH generation was measured. Data shown are means ± SD (n = 3).*, *p* < 0.05 compared with untreated control. Panel **D***,* MCF-7 cells were treated with indicated concentration of Dox, cells were lysed and Western blot analysis of Par-4 and PARP were carried out. Actin was used as a loading control. Panel **E***,* MCF-7 cells were transiently transfected with control siRNA (Cont siRNA) and Par-4 siRNA. After 24 h, cells were treated with either vehicle or 3 μM Dox for further 24 h. The expression of Par-4 and PARP were detected by using Western blot analysis. Actin was used as the protein loading control. Panel **F***,* MCF-7 cells were transiently transfected with Cont siRNA and Par-4 siRNA. After 24 h cells were treated with either vehicle or 3 μM Dox for further 24 h. The cell viability assay was performed. Data shown are means ± SD (n = 3). *, *p* < 0.05, and **, *p* < 0.05 compared with untreated control and control siRNA Dox treated cells, respectively.

To investigate whether Par-4 cleavage is a general feature of human cells undergoing apoptosis, we used different human cancer cell lines and a variety of well-characterized inducers of apoptosis. Jurkat, MCF-7, and LNCaP cells were treated with Doxorubicin (Dox), Etoposide (Eto), and Curcumin (Cur). In all the cases, the treatment resulted in Par-4 cleavage (Figure 2A, *top panel*), followed by induction of apoptosis as evidenced by PARP cleavage (Figure 2A, *middle panel*), and loss of viability (Figure 2B). These experiments allow us to conclude that Par-4 is selectively cleaved during apoptosis, irrespective of the inducing agents and the cell lines under investigation. Hence, suggesting that Par-4 cleavage is an important and may well be a universal step in the apoptotic cell death pathway.

Next, we checked the role of intracellular SPH generation in caspase mediated Par-4 cleavage and apoptosis induction using MCF-7 cells as model system. MCF-7 cells were treated with increasing concentration of Dox for 24 h and endogenous SPH levels, Par-4 cleavage and PARP cleavage were measured. As shown in the Figure 2C and 2D, Dox induced dose dependant SPH generation, Par-4 cleavage and PARP cleavage in MCF-7 cells. The Dox-dependant cleavage of Par-4 and PARP are in line with accumulation of intracellular SPH generation. It is well established that SPH can only be generated from Cer by the action of ceramidase enzyme [31]. In order to confirm that SPH is responsible for the apoptotic induction in MCF-7 cells, cells were pre-treated with acid ceramidase inhibitor N-oleoylethanolamine (NOE) and neutral ceramidase inhibitor D-erythro-2-(N-myristoylamino)-1-phenyl-1-propanol (dMAPP) following treatment with doxorubicin. Inhibition of ceramidase enzymes using both of these inhibitors did not confer protection from apoptosis (data not shown). This may be because of pro-apoptotic action of Cer that will get accumulated when SPH generation is inhibited using ceramidase inhibitors. This is further confirmed by decrease in SPH generation and increase in Cer generation in MCF-7 cells following pre-treatment with these inhibitors (data not shown).

**Figure 3:**
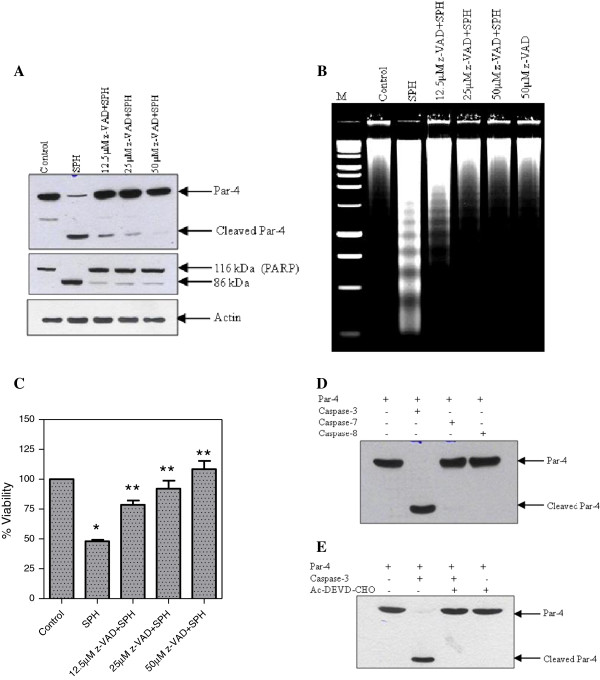
**Cleavage of Par-4 by caspase-3 *in vitro*.** Panel **A***,* Effect of pan caspase inhibitor on the cleavage of Par-4. Jurkat cells were pre-treated with z-VAD-fmk at the indicated concentration for 1 h. The cells were then treated with SPH (8 μM) for 6 h and the lysate were immunoblotted with Par-4 and PARP antibodies. Actin was used as the loading control. Panel **B***,* Jurkat cells were pre-treated with z-VAD-fmk at the indicated concentration for 1 h, then the cells were treated with SPH (8 μM) for 6 h and DNA fragmentation was analysed. Panel **C***,* Jurkat cells were pre-treated with z-VAD-fmk at the indicated concentration for 1 h, then the cells were treated with SPH (8 μM) for 6 h and cell viability was measured by using WST viability assay kit. Data shown are means ± SD (n = 3). *, *p* < 0.05, and **, *p* < 0.05 compared with untreated control and SPH treated cells, respectively. Panel **D***,* Immuno purified Par-4 was incubated with 300 ng of each purified caspase-3, -7, and −8 for 2 h. The reaction was stopped by adding SDS-PAGE sample buffer and proteolytic cleavage of Par-4 was detected by using anti-Par-4 antibody. Panel **E***,* Immuno purified Par-4 was incubated with 300 ng of purified caspase-3 in the presence and absence of caspase-3 specific inhibitor (Ac-DEVD-CHO). The reaction was stopped by adding SDS-PAGE sample buffer and proteolytic cleavage product of Par-4 was detected by using anti-Par-4 antibody.

Next, to check whether Par-4 could be involved in apoptotic induction in response to Dox, MCF-7 cells were transiently transfected with control siRNA or Par-4 siRNA. After 24 h, cells were switched to fresh medium containing 3 μM Dox for further 24 h and whole cell lysates were prepared and analyzed for the expression of Par-4 and PARP. As shown in the Figure 3E, the expression of Par-4 was not affected in the cells transfected with control siRNA where as, the amount of Par-4 is kept at extremely low level in cells transfected with Par-4 siRNA. PARP cleavage analysis and cell viability assay revealed that siRNA mediated knock down of Par-4 significantly reduces the PARP cleavage and loss of viability in response to Dox treatment as compared with that of control siRNA. These data clearly suggest the critical role of Par-4 in induction of apoptosis.

### Caspase-3 is responsible for cleavage of Par-4 during SPH-induced apoptosis

In order to investigate the involvement of caspase family of proteases in the Par-4 cleavage, Jurkat cells were pre-treated for 1 h with different concentrations of pan caspases inhibitor z-VAD-fmk, and the cells were then treated with SPH for further 6 h. As shown in the Figure 3A, z-VAD-fmk, dose dependently suppressed SPH-induced Par-4 cleavage (*top panel*), PARP cleavage (*middle panel*), DNA fragmentation (Figure 3B), and loss of viability (Figure 3C). These data strongly suggest that Par-4 breakdown is due to the apoptotic cysteine protease enzymatic activity mediated by caspase.

Caspase-3, 6, and 7 are the executioners of apoptosis. Amongst them, caspase-3 is the one involved in the cleavage of majority of substrates examined to date [32]. We asked, whether Par-4 could be cleaved by recombinant caspases *in vitro,* and if so, whether, the pattern of the *in vitro* cleavage corresponds to the one observed *in vivo*. To explore the possibility of caspases-mediated cleavage of Par-4 proteins, DDK-tagged Par-4 proteins were immunoprecipitated from HEK-293 cell lysates after transient transfection. The precipitated proteins were incubated with active recombinant caspases-3, -7 or −8. As shown in Figure 3D, caspases-3 efficiently cleaved Par-4, while caspases-7 and 8 did not cleave Par-4 under the condition tested. The caspase-3 inhibitor, DEVD-CHO blocked caspase-3 mediated cleavage of Par-4 (Figure 3E). Notably, the pattern of Par-4 cleavage *in vitro* by recombinant caspases-3 is identical to that observed *in vivo*, resulting in the generation of 24 kDa Par-4 fragment. Because of the similarity between *in vitro* and the *in vivo* Par-4 cleavage pattern, it is very likely that caspase-3 is the responsible protease for the Par-4 cleavage *in vivo* in SPH-induced apoptosis in Jurkat cells.

Interestingly, in the present study Par-4 cleavage has also seen in the MCF-7 cells (Figure 2A), which were previously reported to be lacking caspase-3 activity due to the functional deletion of *CASP-*3 gene [33]. The possible explanation for this cleavage is the existence of yet unidentified caspase-3-like proteases in MCF-7 cells which can compensate for the lack of caspase-3. This yet unidentified caspase-3 like protease has also been reported to be responsible for the cleavage of proteins such as PARP, Rb, PAK2, DNA-PK, gelsolin and DFF-45 in the MCF-7 cells [34]. All these aforementioned proteins are well established substrates for caspase-3 in other cell lines [34-37].

### Cleavage of Par-4 leads to the release of SAC domain containing fragments from Par-4

**Figure 4:**
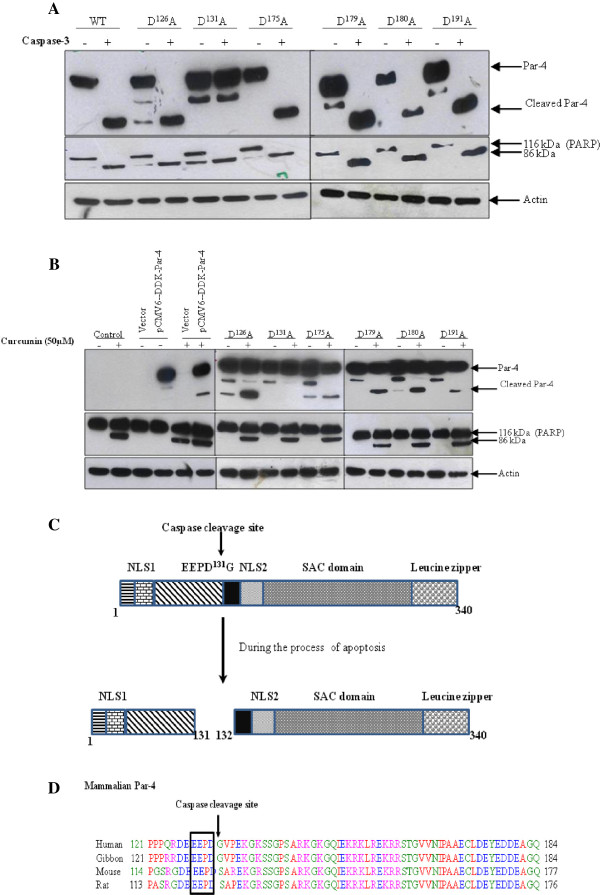
**Identification of the cleavage site.** Panel **A***,* D^126^A, D^131^A, D^175^A, D^179^A, D^180^A, and D^191^A mutant were constructed. Each mutants were transiently transfected into HEK-293 cells. Cells were harvested 1 day after transfection and the cytosolic fraction of each transfectants were incubated with caspase-3 for 2 h *in vitro*. The proteolytic cleavage product of Par-4 was detected by anti-DDK monoclonal antibody. The activity of caspase-3 was also confirmed by checking the PARP cleavage. Actin was used as the loading control. Panel **B***,* Wild type and each mutant Par-4 was transiently transfected in to HEK-293 cells and the cells were treated with either vehicle or 50 μM Cur for 24 h. The proteolytic cleavage of each mutant Par-4 was detected by immuno-probing of each lysate with anti-DDK monoclonal antibody. Apoptosis induction was confirmed by PARP cleavage and actin was used as protein loading control. Panel **C***,* Schematic representation of the release of SAC domain containing fragment from the Par-4 during the apoptosis. Nuclear localization sequences (NLS1 and NLS2), selective for apoptosis induction in cancer cells (SAC). Panel **D***,* Alignments of the regions containing the caspase-3 cleavage site of Par-4 from different species.

*In silico* analysis of Par-4 protein sequence did not reveal the classical caspase recognition tetrapeptide sequence (DEXD). We found six possible cleavage sites; PQRD^126^, EEPD^131^, ECLD^175^, EYED^179^, YEDD^180^ and KRED^191^. In order to identify whether any of these six putative cleavage site is cleaved by caspase-3, we mutated aspartatic acid (D) to alanine (A) in Par-4. These mutants were tagged at carboxy-terminus with DDK epitope. These six mutants, as well as the wild-type Par-4, were transiently expressed in HEK-293 cells, and cell extracts were incubated with the active caspases-3. The cleavage of the wild-type and mutants were detected by probing with anti-DDK monoclonal antibody. The D^126^ → A, D^175^ → A, D^179^ → A, D^180^ → A and D^191^ → A mutants were cleaved by caspase-3 and generated 24 kDa cleavage product (Figure 4A, *top panel*). However, the D^131^ → A mutant completely blocked the cleavage by caspase-3 (Figure 4A, *top panel*). Notably, PARP, a well known substrate for caspase-3, was effectively cleaved in all mutants of Par-4 (Figure 4A, *middle panel*). Furthermore, the D^131^ → A mutant was resistant to cleavage by caspases during curcumin-induced apoptosis in HEK-293 cells (Figure 4B, *top panel*). Neither a significant decrease in the amount of intact protein, nor an increase in the cleavage product was observed in the D^131^ → A mutant. If Par-4 cleaves at D^131^, it will generate NH_2_-terminal (Amino acids 1–131, ~16 kDa), and a carboxy-terminal (amino acid 132–340, ~24 kDa) fragments (Figure 4C). The size of the fragments detected by anti-DDK antibody, which recognizes carboxy-terminal end of the expressed Par-4 correlated with the predicted fragment size. It has been reported that Par-4 contains a unique core domain (amino acids 137–195), which when over-expressed, induces apoptosis significantly in cancer cells, including those that were resistant to full length Par-4 mediated apoptosis. This domain is referred to as “selective for apoptosis induction in cancer cells” (SAC) domain [8]. This segment also contains NLS2 domain, which facilitates its nuclear translocation [8]. Interestingly, cleavage of Par-4 at amino acid residue D^131^ separates the SAC domain containing carboxy-terminus from rest of the protein (Figure 4C). The cleavage site is conserved in higher eukaryotes from human to rat (Figure 4D). Taken together, our data demonstrate the presence of a caspase-3 cleavage site in Par-4 at residue D^131^, the cleavage of which leads to the release of SAC domain containing fragment from the Par-4 during the process of apoptosis.

### Akt phosphorylation at Ser^473^ regulates caspase-dependant cleavage of Par-4 during SPH-induced apoptosis in Jurkat cells

**Figure 5:**
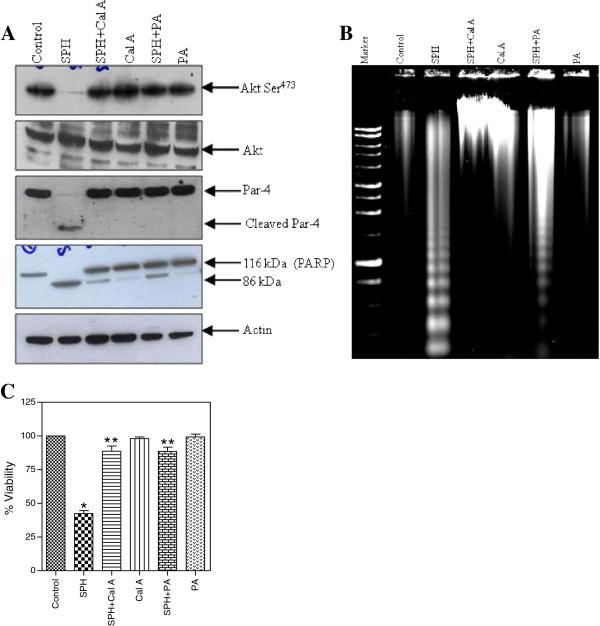
**Inhibition of Akt dephosphorylation protects from SPH-induced Par-4 cleavage and apoptosis.** Panel **A***,* Jurkat cells were pre-treated with 5 nM calyculin A (Cal A) and 30 μM phosphatidic acid (PA) followed by treatment with 8 μM SPH. Cells were lysed and fractionated by SDS-PAGE. Western blot were probed with antibodies specific for phospho-Akt (Ser^473^), Akt, Par-4 and PARP. Actin was used as loading control. Panel **B***,* Jurkat cells were pre-treated with 5 nM Cal A and 30 μM PA followed by treatment with 8 μM SPH and DNA fragmentation analysis was performed. Panel **C**, Jurkat cells were pre-treated with 5 nM CalA and 30 μM PA followed by treatment with 8 μM SPH and viability assay was done by using WST assay kit. Data shown are means ± SD (n = 3). *, *p* < 0.05, and **, *p* < 0.05 compared with untreated control and SPH treated cells, respectively.

Previously, we have shown that protein phosphatase-1 (PP1) activation, and dephosphorylation of Akt is the prime signalling event in SPH-induced apoptosis in Jurkat cells [12]. Hence, we examined whether the suppression of SPH-induced Akt dephosphorylation has any relevance to SPH-induced Par-4 cleavage. In order to achieve this, we used two potent inhibitors of protein phosphatase-1 (calyculin A and phosphatidic acid). As shown in the Figure 5A, both calyculin A and PA prevented SPH-induced Akt dephosphorylation, Par-4 cleavage and PARP cleavage. Additionally, the effect of pre-treatment with calyculin A and PA on DNA damage and cell viability were also examined. As shown in Figure 5B and 5C, pre-treatment of Jurkat cells with calyculin A and PA significantly abrogated SPH-induced DNA damage and loss of viability. This data clearly indicate that SPH-induced caspase dependant cleavage of Par-4 in Jurkat cells is strictly under the control of Akt phosphorylation at Ser^473^.

## Discussion

Completion of apoptosis requires highly selective degradation of some proteins by caspases [29]. Identification of caspase substrates is important to identify and understand the underlying molecular mechanism critical to apoptosis induction [32]. We have shown that Par-4 is specifically cleaved by caspase-3 to produce SAC domain containing fragment during SPH-induced apoptosis in Jurkat cells. In order to study the particular role of SPH in apoptosis, cell death should be measured after exogenous administration of SPH. But, there is a possibility that, cells when treated with SPH, it can be converted to another pro-apoptotic lipid, Cer by the action of an enzyme Cer synthase. In one of our previously published data, we have shown that pre-treatment of Jurkat cells with fumonisin B1 (FB1), a known inhibitor Cer synthase protected the cells from SPH mediated Cer generation [12]. However, FB1 did not protect the Jurkat cells from SPH mediated apoptosis induction. This data clearly suggests that, SPH treatment itself is capable of inducing apoptosis in Jurkat cells; not by converting it in to Cer.

The cleavage of Par-4 may be significant since it is induced by diverse apoptosis-inducing agents in different cancer cell lines. The fact that Par-4 cleavage, PARP cleavage, and apoptosis induction were inhibited by a cell permeable caspase inhibitor z-VAD-fmk indicates that caspases may well be involved in the proteolytic cleavage of Par-4. Three lines of evidence support the assumption that caspase-3 is responsible for the release of SAC domain containing fragment from Par-4 during apoptosis. First, recombinant active caspase-3 cleaves the immunoprecipitated Par-4; second, a specific tetrapeptide inhibitor of caspase-3, DEVD-CHO efficiently blocks Par-4 cleavage *in vitro*; third, mutation of the cleavage site (D^131^ → A) prevents Par-4 cleavage both *in vitro* and *in vivo*.

Caspases have unique requirement for aspartic acid in the P1 position of peptide substrates, with their selectivity being partially dependent on the amino acids at position P4, and to a lesser extent, at P2 and P3 [38]. The typical caspase-3 consensus site is believed to require an aspartate residue at P4 position (DEXD) [39]. There is no DEXD motif in the Par-4 sequence. However, numerous exceptions to this rule have been reported [20]. For example, both PLC-γ1 [40] and topoisomerase I [41] are cleaved by caspase-3 at the unconventional sequences AEPD^770^ and EEED^170^ respectively. We have identified six possible cleavage sequences for Par-4: PQRD^126^, EEPD^131^, ECLD^175^, EYED^179^, YEDD^180^ and KRED^191^. Although, the P4 position of the cleavage site is Glu instead of Asp (i.e. E instead of D), EEPD^131^ is the cleavage site recognized by caspase-3 *in vitro*. Since size of the cleavage product is the same in both *in vitro* and *in vivo*, EEPD sequence could be the cleavage site for caspase-3 in Par-4. Moreover, our results are principally consistent with a study published recently by Chaudhry and colleagues [30].

Par-4 contains two putative nuclear localization sequences (NLS), designated as NLS1 (amino acid 20–25) and NLS2 (amino acid 137–153) in the N-terminal region, leucine-zipper domain (amino acids 290–332), and a nuclear export sequence in the C-terminus [8]. The protein also possesses several consensus phosphorylation sites for kinases, such as protein kinase A (PKA) and Akt [19,42]. These domains are considered to be critical in regulating the function of Par-4 protein. Analysis of several mutants, resulting from serial deletion of both N-terminus and C-terminus residues from the full length Par-4 protein, lead to identification of a unique core domain (amino acid 137–195). This core domain, when overexpressed, induces apoptosis specifically in cancer cells, and therefore, this domain is referred to as “selective for apoptosis induction in cancer cells” (SAC) domain [8]. This segment contains NLS2 (amino acid 137–153) domain which facilitate its nuclear translocation. It has been reported that the cancer selective apoptotic action of Par-4 requires two distinct events; specifically, nuclear entry and phosphorylation by PKA at Thr^155^[42]. Both regulating events are present and active in cells that display sensitivity to Par-4 (Par-4- susceptible cells). However, cells that are resistant to Par-4, such as hormone-dependant cancer cells like LNCaP and MCF-7 cells, are unable to localize the full length Par-4 to the nucleus. Despite the presence of substantial amounts of Par-4 and elevated PKA activity, the cells remain resistant to apoptosis. However, it has been reported that SAC domain can enter in to the nucleus, and it induces apoptosis in both Par-4 susceptible and Par-4 resistant cancer cells, regardless of whether they are of prostate origin or not [8]. Therefore, caspase-3-mediated cleavage of Par-4 might be a mechanism to modulate its apoptotic function. One possible consequence of Par-4 cleavage is that it represents a mechanism to activate Par-4-mediated apoptosis in Par-4 resistant cancer cells. Since the cleavage site EEPD^131^ is adjacent to the SAC (amino acid 137–195) domain, the cleavage of Par-4 by caspase-3 may very well be the mechanism to release the SAC domain from Par-4, which may be sufficient to induce or augment apoptosis in Par-4-resistant and susceptible cancer cells.

Akt is a key downstream effector of PI3-Kinase that blocks apoptosis in a variety of cell types [16]. Akt is activated when cells are exposed to growth factors, and its activation occurs via a pathway that includes PI3-kinase activation [16]. Previously, we have reported that SPH induces PP1-dependant rapid dephosphorylation of Akt that leads to caspase activation and apoptosis in Jurkat cells [12]. It has also been reported that Akt physically binds to the pro-apoptotic protein Par-4 via its leucine zipper domain, and phosphorylates Par-4 to inhibit apoptosis [19]. The present study also demonstrates that inhibition of SPH induced Akt dephosphorylation attenuates caspase dependant cleavage of Par-4 and apoptosis. This suggests a critical role of Akt in SPH-induced Par-4 cleavage and apoptosis. However, further studies are required to elucidate the additional role Akt dephosphorylation and Par-4 cleavage in the regulation of apoptosis.

In conclusion, evidence provided here shows that Par-4 is cleaved during SPH-induced apoptosis by caspase-3. The present study has explored a new, yet-unexplained apoptotic mechanism of SPH through a novel post translational modification of Par-4. However, complete understanding of the interplay between Par-4 and caspases and their mechanisms based on signal transduction still requires much more study.

## Materials and methods

### Antibodies and reagents

Anti-Par-4 (C-19, A-10, and R-334), anti-actin, and anti-goat IgG were obtained from Santa Cruz Biotechnology (CA, USA). Anti-PARP, anti-Akt, and anti-phospho Akt (Ser^473^) antibodies were from Cell Signaling Technology (Beverly, MA, USA). D-erythro-sphingosine from Avanti Polar Lipids, Inc. CaspASE assay system colorimetric from Promega (Madison, USA). WST-8 cell counting kit, Ac-DEVD-CHO and z-VAD-fmk were purchased from Alexis. Anti-rabbit IgG, anti-mouse IgG, doxorubicin, etoposide, curcumin, phosphatidic acid and all other fine chemicals were obtained from Sigma chemicals Co (St. Louis, MO, USA). Anti-DDK antibody and Mega Tans 1.0 were purchased from OriGene Technologies, Inc. Calyculin A was from Calbiochem (La Jolla, CA, USA).

### Cell culture and drug treatment

Jurkat (Acute lymphocytic T-cells leukemia), MCF-7, and LNCaP (ATCC, Rockville, MD) were grown in RPMI 1640 containing GlutaMAX medium and HEK-293 cells were grown in DMEM medium in humidified atmosphere of 95% air and 5% CO_2_ at 37°C. Both the medium were supplemented with 10% (V/V) heat inactivated FBS without antibiotics. Cell culture regents were obtained from Gibco-BRL. For the induction of apoptosis, Jurkat cells were treated with 0–8 μM SPH for various times interval or with 0.2 μM Dox or 4 μM Eto or 25 μM Cur for 24 h. The MCF-7 cells were treated with 3 μM Dox or 100 μM Eto or 40 μM Cur and LNCaP cells were treated with 5 μM Dox or 50 μM Eto or 50 μM Cur for 24 h. Caspase-3 inhibitor (Ac-DEVD-CHO) and pan-caspases inhibitor (z-VAD-fmk) were added to the culture medium 1 h prior to the treatment. Inhibitors were used according to the manufactures instructions.

### Plasmids, siRNA and transient transfection

pCMV6-XL6-Par-4 (SC110969) and pCMV6-Myc-DDK-tagged PAR-4 (RC202733) were purchased from OriGene Technologies, Inc. DNA transfection to HEK-293 cells was performed by using Mega Tans 1.0 transfection reagent as described in the manufacture’s protocol. To knockdown the endogenous Par-4, cells were transiently transfected with 10 nM of siRNAs targeting Par-4 (SC#36190 from Santa Cruz biotechnology, USA) or with the non-silencing control siRNA (SC#37007 from Santa Cruz Biotechnology, USA) using HiPerFect (Qaigen) transfection reagent according to the manufacturer’s recommendations.

### Generation of caspases-resistant mutant Par-4

The mutant clones were generated by site-directed mutagenesis of a pCMV6-Myc-DDK-tagged PAR-4 (RC202733) purchased from OriGene Technologies, Inc. The mutations were confirmed by sequencing analysis.

### Western blotting

Cells were washed twice with phosphate buffered saline (PBS) and lysed in a RIPA lysis buffer [50 mM Tris HCl (pH 7.4), 1% NP-40, 40 mM NaF, 10 mM NaCl, 10 mM Na_3_VO4, 1 mM phenylmethylsufonyl fluoride (PMSF) and 10 mM dithiothreitol (DTT) and EDTA-free protease inhibitor tablets per 20 ml buffer]. The cell lysates were centrifuged at 14000 rpm for 15 min. Total protein, determined by Bio-Rad protein assay, were mixed with 6X loading buffer and boiled at 100°C for 3 min. Samples at 40 μg/lane were resolved by SDS-PAGE and the separated proteins were transferred on to nitrocellulose membrane by wet transfer method using Bio-Rad electro transfer apparatus. Following transfer, the blots were blocked with 5% non-fat milk in tris-buffer saline containing 0.1% Tween-20. Blots were then incubated with primary antibodies followed by secondary antibody. Proteins were visualized using enhanced chemiluminescence system.

### Measurement of Par-4 cleavage

To determine the cleavage of Par-4, 50 μg of whole cell extract was resolved on 10% polyacrylamide gel, transferred to nitrocellulose membrane, blocked with 5% non-fat milk protein, probed with Par-4 antibody (1:1000) followed by secondary antibody. The Par-4 protein and cleaved fragment were detected by using enhanced chemiluminescence reagent (Pierce Biotech, Rockford, IL).

### In Vitro Par-4 cleavage assay

Anti-DDK antibody covalently immobilized with AminoLink Plus coupling resin using direct immunoprecipitation kit (Pierce Biotechnology, Rockford, IL). The immunoprecipitated DDK-Par-4 from HEK-293 cells lysate was incubated with active recombinant caspases in reaction solution containing 50 mM Hepes (pH 7.4), 50 mM NaCl, 5% glycerol, 0.1% CHAPS, 10 mM EDTA and 10 mM DTT at 37°C for 2 h. The reaction was terminated by the addition SDS-loading buffer to the reaction mixture. The sample were resolved on an SDS-PAGE gel and analysed by western blotting.

### Enzymatic caspases-3 assay

The enzymatic assay of caspase induced by SPH was measured by using the manufacture’s protocol (Promega). Briefly, cells were lysed in a lysis buffer by freeze and thawing. The lysed cells were centrifuged at 14000 rpm for 15 min. 50 μg of protein was incubated with 30 μl of caspase assay buffer and 2 μl of caspase-3 (DEVD-pNA) colorimetric substrate at 37°C for 4 h. The optical density of the reaction mixture was quantitated spectrophotometrically at a wavelength of 405 nm by using 96 well plate reader (Perkin Elmer spectrofluorometer, Victor X3).

### Cell viability assays

Cell viability was evaluated with WST-8 [2-(2-methoxy-4-nitrophenyl)-3-(4-nitrophenyl)-5-(2,4-disulfophenyl)-2H-tetrazolium, monosodium salt] assay kit according to the manufacturer’s instruction. WST-8 solution was added at 1:100 in culture medium. After 2 h incubation, the light absorbance (test wavelength OD 450 nm, reference wavelength 655 nm) was measured using 96 well plate reader (Perkin Elmer spectrofluorometer, Victor X3). The cytotoxicity was expressed as percentage over control.

### Intracellular SPH measurement

Cells were washed in PBS and lysed 50 mM Tris (pH-7.4) containing 0.4% IGEPAL CA 630 by freeze and thaw method. The final concentration of IGEPAL CA 630 in the assay was 0.2%. The lysate were then heat at 70°C for 5 min in a water bath and centrifuged at 12000 rpm for 10 min at 4°C. The released SPH was derivatized with *o*-phthaladehyde (OPA) reagent as described previously [12]. An aliquot of 25 μl was used for the SPH analysis. HPLC analysis was done using *Waters 1525* binary pump system. Waters XTerra RP18 (5 μm, 3 mmx250 mm) column was equilibrated with a mobile phase (20% methanol, 80% 1:9, 0.07 M potassium hydrogen phosphate buffer: methanol) at a flow rate of 0.5 ml/min. The fluorescence detector (*Waters 2475*) was set at an excitation wavelength of 340 nm and an emission wavelength of 455 nm.

### DNA fragmentation analysis

Apoptotic DNA fragments were isolated from the apoptotic cells as described previously [43]. After treatment cells were washed with PBS and incubated with 200 μl of lysis buffer (50 mM Tris–HCl (pH 7.5), 3% non-ionic detergent IGPAL CA-630 [(Octylphenoxy) polyethoxyethanol] and 20 mM EDTA) for 10 min. The samples were centrifuged at 1000xg for 5 min in order to collect the supernatant which contain apoptotic DNA fragment. Sodium dodecyl sulfate (SDS) (10 μl, 20%) was added and the supernatants were incubated with 0.4 μg/ml RNase at 56°C for 2 h to remove the cellular RNA. Proteinase K (1.5 μg/ ml) was then added to the supernatant at 56°C and it was further incubated for 2 h to remove the proteins. The DNA was then precipitated with 0.1 volume of 3 M sodium acetate and 2.5 volume of ice cold absolute ethanol. After centrifugation, the DNA pellet was washed with 70% ethanol and then air dried. The dried pellet was re-suspended in 20 μl TE buffer (10 mM Tris–HCl, pH 7.5 and 0.1 mM EDTA) and incubated at 65°C for 5 min. Finally the resuspended DNA was subjected to electrophoresis on a 2% agarose gel at a constant voltage of 40 V for 1–2 h.

### Statistical analysis

Data are presented as the mean ± standard deviation (SD) from three independent experiments. Statistical analysis was conducted by using unpaired *t*-tests. A *p* value of < 0.05 was considered statistically significant.

## Abbreviations

SAC: Selective for apoptosis induction in cancer cells; SPH: Sphingosine; Cur: Curcumin; Dox: Doxorubicin; DTT: Dithiothreitol; ETO: Etoposide; NLS: Nuclear localization sequences; Par-4: Prostate apoptotic response-4; PARP: Poly (ADP-ribose) polymerase; PMSF: Phenylmethylsufonyl fluoride; Cal A: Calyculin A; PA: Phosphatidic acid.

## Competing interests

The author(s) declare that they have no competing interests.

## Authors’ contributions

SG designed the study and analyzed the data. FT carried out the experiments and drafted the manuscripts. SP, AR, JK, SC and MP provided support and coordination, editing various drafts of the manuscript. All authors read and approved the final manuscript.
